# Comprehensive profiling of lysine acetylproteome analysis reveals diverse functions of lysine acetylation in common wheat

**DOI:** 10.1038/srep21069

**Published:** 2016-02-15

**Authors:** Yumei Zhang, Limin Song, Wenxing Liang, Ping Mu, Shu Wang, Qi Lin

**Affiliations:** 1 College of Agronomy, Shenyang Agricultural University, Shenyang, Liaoning 110866, China; 2 College of Agronomy and Plant Protection, Qingdao Agricultural University, Qingdao, Shandong 266109, China

## Abstract

Lysine acetylation of proteins, a dynamic and reversible post-translational modification, plays a critical regulatory role in both eukaryotes and prokaryotes. Several researches have been carried out on acetylproteome in plants. However, until now, there have been no data on common wheat, the major cereal crop in the world. In this study, we performed a global acetylproteome analysis of common wheat variety (*Triticum aestivum* L.), Chinese Spring. In total, 416 lysine modification sites were identified on 277 proteins, which are involved in a wide variety of biological processes. Consistent with previous studies, a large proportion of the acetylated proteins are involved in metabolic process. Interestingly, according to the functional enrichment analysis, 26 acetylated proteins are involved in photosynthesis and Calvin cycle, suggesting an important role of lysine acetylation in these processes. Moreover, protein interaction network analysis reveals that diverse interactions are modulated by protein acetylation. These data represent the first report of acetylome in common wheat and serve as an important resource for exploring the physiological role of lysine acetylation in this organism and likely in all plants.

Post-translational modifications (PTMs) frequently occur to proteins during or after protein biosynthesis, and play an important role in modulating diverse cellular processes^1^. PTMs can change protein functions by introducing new functional groups such as phospho, acetyl, ubiquityl and methyl groups. Among them, acetylation of lysine is a highly dynamic and reversibly regulated PTM, which changes protein function in multiple ways^2^. As one of the most common PTMs to proteins in both eukaryotes and prokaryotes^2^,^3^, lysine acetylation occurs on either the α-amino group at the N-terminus of the protein or the ε-amino group on the side chain of lysine residues^4^.

Lysine acetylation was first discovered in histone proteins^5^,^6^ and its role has been extensively investigated in regulating gene transcription^7^,^8^,^9^. In addition to histones, many other proteins in nucleus, cytoplasm and mitochondria were also found to be acetylated^10^. Previous studies indicated that protein acetylation regulates a wide variety of important cellular processes, such as enzymatic activity^11^,^12^, protein interactions^2^, protein stability^13^ and metabolic pathways^3^.

Advances in liquid chromatography−mass spectrometry (LC−MS/MS) and lysine-acetylated peptide immunoprecipitation enable lysine acetylation to be investigated on a proteomic scale^14^. In recent years, a large number of lysine-acetylated proteins have been identified in both microbes and mammalians. These proteome-wide analyses of lysine acetylation revealed its broad role in various cellular functions. However, compared to these organisms, the acetylome of plants is poorly studied^15^.

Common wheat (*Triticum aestivum* L.), also known as bread wheat, is one of the most important cereal crops in the world. Chinese Spring, characterized by its easy hybridization, high yielding and wide adaptability, is a specific white wheat variety in Sichuan Basin of China and has been internationally known as one of the most important wheat germplasm resources in China. The presence of a large number of acetyltranferase and deacetylase orthologs in the wheat genome suggests that lysine acetylation of proteins may play a critical role in wheat development and metabolism. In this paper, we report a proteomics study of lysine acetylation in common wheat variety, Chinese Spring. We identified 416 acetylated sites on 277 proteins controlling various biological processes in this plant. These acetylated proteins are localized in multiple compartments including nucleus, cytosol, chloroplast, mitochondria, cytoskeleton, extracellular and plasma membrane. Importantly, 26 proteins involved in photosynthesis and Calvin cycle are found to be acetylated. These results provide a systems-wide view of the wheat acetylome and an affluent dataset for functional analysis of acetylated proteins in this important cereal crop.

## Results

### Proteome-wide analysis of lysine acetylation sites and proteins in wheat

**Figure 1:**
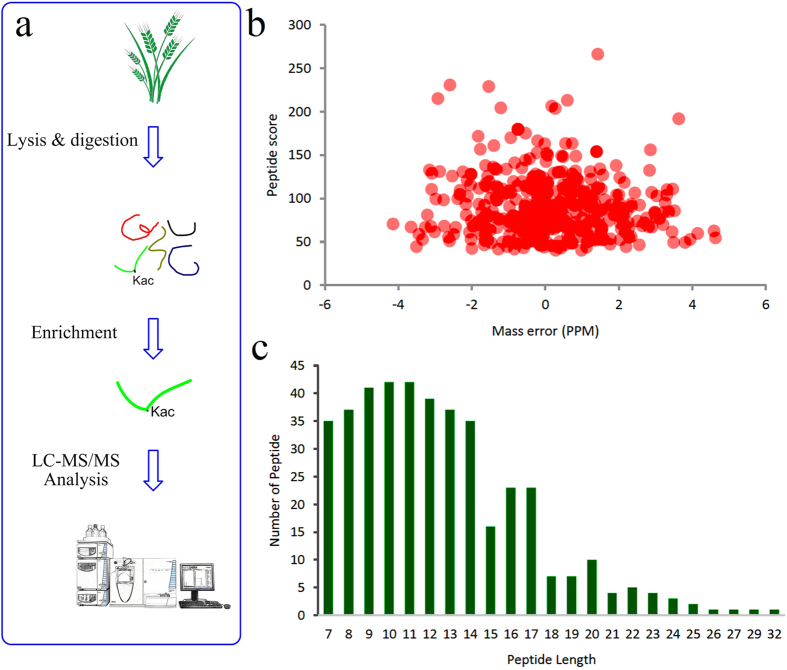
Proteome-wide identification of lysine acetylation sites in wheat. (**a**) Overview of experimental procedures used in this study. (**b**) Mass error distribution of all identified peptides. (**c**) Peptide length distribution.

Overview of experimental procedures used in this study was shown in Fig. 1a. To map lysine acetylation sites in wheat, proteins were extracted and digested into peptides with trypsin as described. Then, acetylated peptides were immune-enriched and analyzed using high-resolution LC-MS/MS. The mass errors of all the identified peptides were checked and most of them were less than 0.02 Da, confirming the high accuracy of the MS data (Fig. 1b). The length of most acetylation peptides obtained above was distributed between 7 and 32, which is consistent with the property of tryptic peptides (Fig. 1c). Altogether, 416 lysine acetylation sites in 277 proteins were identified (supplementary Table S1). The acetylome of wheat was compared to those of other plants reported, and the results were presented in Table 1. Notably, wheat has a large quantity of acetylated proteins among the plants studied so far, reflecting a potentially crucial role of this post-translational modification in the plant.

### Distribution and motif analysis of lysine acetylation sites

**Figure 2:**
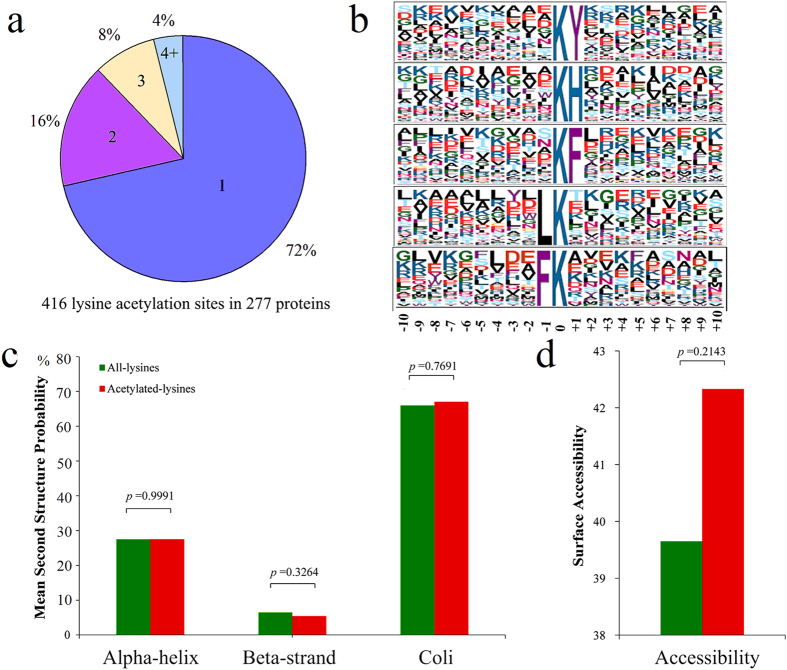
Properties of lysine acetylation sites. (**a**) Pie chart illustrating the number and percentage of lysine acetylation sites per protein. (**b**) Sequence probability logos of significantly enriched acetylation site motifs for ±10 amino acids around the lysine acetylation sites. (**c**) Probabilities of lysine acetylation in different protein secondary structures (alpha-helix, beta-strand and coli). (**d**) Predicted surface accessibility of acetylation sites. All lysine sites were in green and acetylated lysine sites were in red.

In order to assess the distribution of acetylation sites in the acetylated proteins of wheat, the numbers of identified modification sites per protein were calculated. The results indicated that 72% of proteins contained only one acetylation site, and the percentage of proteins with two, three and four or more modification sites were 16%, 8% and 4%, respectively (Fig. 2a).

To understand the acetylation motifs in wheat, the context of amino acid sequence surrounding the acetylated lysines was analyzed. Substantial bias in amino acid distribution was observed from position −10 to +10 around the acetylated lysines in the 416 peptides identified using the motif-x program. Five conserved sequences around acetylation sites, namely, K^AC^Y, K^AC^H, K^AC^F, LK^AC^ and FK^AC^, were found in the wheat acetylproteome (Fig. 2b, Supplementary Table S2, Supplementary Table S3). A survey of these motifs indicated that enrichment of a residue with hydrophobic side chain groups, such as tyrosine (Y) and phenylalanine (F), was observed at the +1 position. A positively charged residue, histidine (H), was also found to be enriched in the +1 position. Furthermore, two kinds of amino acids with hydrophobic side chain groups, leucine (L) and phenylalanine (F), were enriched in the −1 position (Fig. 2b). These results suggest that amino acid residues with positive charge and hydrophobic side chain might be functionally important for acetylation to occur.

To elucidate the relationship between lysine acetylation and protein structure, the local secondary structures of acetylated proteins was investigated (Fig. 2c). The results indicated that 32.95% of the acetylation sites was located at regions with ordered secondary structure. Among them, 76 sites were located in α-helix and 15 were in beta-strand. In addition, 67.05% of the acetylation sites was located in the disordered structures of proteins. However, based on the similarity of distribution pattern between acetylated lysine and all lysine, it seems there is no tendency of acetylation in wheat proteins. Surface accessibility of the acetylated lysine sites was also evaluated. The results showed that 42.33% of the acetylated lysine sites were exposed to protein surface, compared with 39.65% of all lysine residues (*p* = 0.2143) (Fig. 2d). Therefore, the surface property of proteins is not likely to be changed by lysine acetylation.

### Functional distribution and cellular localization of acetylated proteins in wheat

**Figure 3:**
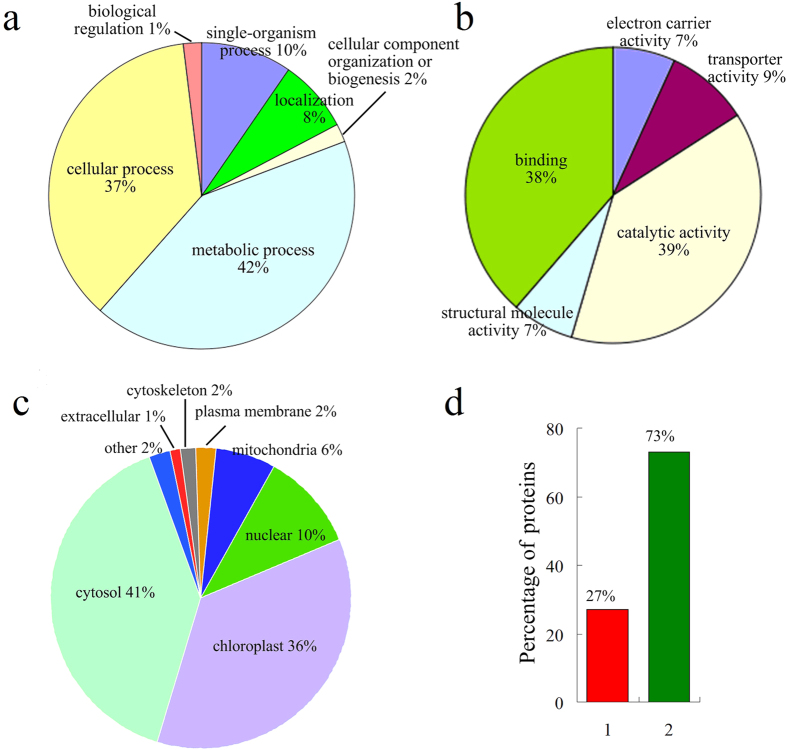
Functional classification of acetylated proteins in wheat. (**a**) Classification of the acetylated proteins based on biological process. (**b**) Classification of the acetylated proteins based on molecular function. (**c**) Subcellular localization of the acetylated proteins. (**d**) Percentage of the acetylated proteins involved in photosynthesis and Calvin cycle (in red), and other processes (in green) in chloroplast.

To better understand the acetylome in wheat, GO functional classification of all the acetylated proteins was investigated based on their biological process, molecular function and cellular component (Fig. 3, Supplementary Table S4). The largest group of acetylated proteins consists of enzymes that are associated with metabolism (42%) in the biological process classification (Fig. 3a). This is not surprising at all since a large proportion of acetylated proteins identified so far was involved in metabolic process. Most acetylated proteins were found to be related to catalytic activity (39%) and binding (38%) in the molecular function classification, and others were assigned to transporter activity (9%), electron carrier activity (7%) and structural molecule activity (7%) (Fig. 3b).

Subcellular localization analysis revealed that most of the identified acetylated proteins in wheat were located to the cytosol (41%) and chloroplast (36%) (Fig. 3c). Further study showed that 27% of the acetylated proteins present in chloroplast were involved in the processes of Calvin cycle and photosynthesis (Fig. 3d). As expected, 10% of the acetylated proteins including histones were localized in the nucleus (Fig. 3c), confirming the regulatory role of lysine acetylation in post-translational regulation. Furthermore, some proteins were predicted to be distributed in the mitochondria (6%), cytoskeleton (2%), plasma membrane (2%) and extracellular space (1%) (Fig. 3c). These data, together with the results of GO functional classification, suggest that the lysine acetylated proteins have a broad range of biological functions in wheat.

### Functional enrichment analysis of acetylated proteins

**Figure 4:**
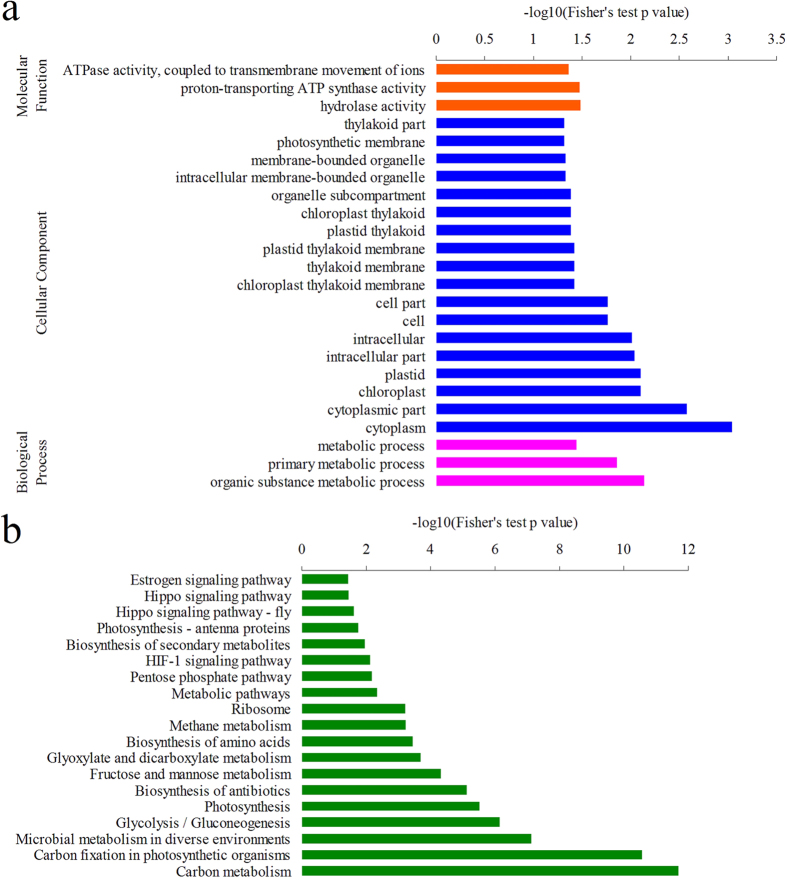
Enrichment analysis of the acetylated proteins in wheat. (**a**) GO-based enrichment analysis of the acetylated proteins in terms of molecular function, cell component and biological process. (**b**) KEGG pathway-based enrichment analysis of the acetylated proteins.

In order to further study biological processes (Supplementary Table S5), molecular functions (Supplementary Table S6) and cellular components (Supplementary Table S7) in which the acetylated proteins were involved, a GO enrichment analysis was performed (Fig. 4a). The results indicated that the acetylated proteins in wheat were significantly enriched in metabolic processes, which is in agreement with the observation that proteins with ATPase activity, proton-transporting ATP synthase activity and hydrolase activity have a higher tendency to be acetylated (Fig. 4a). Consistent with these findings, proteins enriched in cytoplasm, chloroplast and plastid are more likely to be acetylated based on the cellular component analysis (Fig. 4a).

To better understand the general function of these acetylated proteins in wheat, they were mapped to KEGG metabolic pathways (Fig. 4b, Supplementary Table S8). The results showed that lysine acetylation occurs on many proteins involved in carbon metabolism (Supplementary Fig. S1), glycolysis/gluconeogenesis (Supplementary Fig. S2), carbon fixation (Supplementary Fig. S3) and biosynthesis of amino acids (Supplementary Fig. S4).

### Acetylated proteins involved in photosynthesis and carbon metabolism

**Figure 5:**
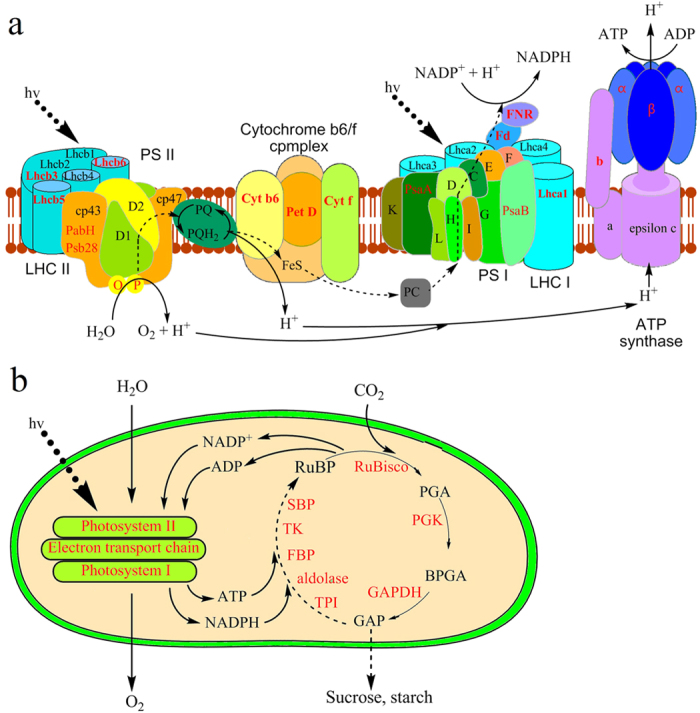
Working scheme of lysine acetylation events involved in Calvin cycle (**a**) and photosynthesis (**b**) in wheat. Identified acetylated proteins were highlighted in red. RuBisco: ribulose bisphosphate carboxylase/oxygenase; PGK: phosphoglycerate kinase; GAPDH: glyceraldehyde 3-phosphate dehydrogenase; TPI: triosephosphate isomerase; FBP: fructose-1,6-bisphosphatase; TK: Transketolase; SBP: Sedoheputulose-1,7-bisphosphatase; LHC I: light-harvesting complex I; LHC II: light-harvesting complex II; PS I: Photosystem I; PS II: Photosystem II; Lhca1~4: light-harvesting complex I chlorophyll a binding protein 1~4; Lhcb1~6: light-harvesting complex II chlorophyll b binding protein 1~6; PQ: plastoquinone; PC: plastocyanin; Fd: ferredoxin; FNR: ferredoxin NADP^+^ reductase.

Functional enrichment analysis of identified acetylated proteins indicated that lysine acetylation may play an important role in photosynthesis. Consistent with this hypothesis, a total of 26 proteins involved in photosynthesis and Calvin cycle were found to be acetylated (Fig. 3d). In higher plants, light-harvesting complex (LHC), a functional unit in photosynthesis, can collect more of the incoming light than would be captured by the photosynthetic reaction center alone. In wheat, four subunits of LHC, Lhca1, Lhcb3, Lhcb5 and Lhcb6, were identified to be acetylated proteins by MS analysis (Fig. 5a).

The cytochrome b6f complex is located in the thylakoid membrane in chloroplasts of plants, catalyzing the transfer of electrons between the two reaction complexes from Photosystem II (PS II) to Photosystem I (PS I). The results of LC-MS/MS analysis indicated that three subunits of cytochrome b6f complex (Cyt b6, Cyt f and Pet D), four subunits of PS II (PsbO, PsbP, PsbH and Psb28) and two ubunits of PS I (PsaA and PsaB) were among the acetylated proteins (Fig. 5a).

Fd (Ferredoxins), one kind of iron-sulfur proteins, mediates electron transfer in a range of metabolic reactions. FNR (Ferredoxin-NADP^+^ oxidoreductase) catalyzes reversible electron transfer between Fd and NAD(P)H^16^. Our data showed that lysine acetylation occurs to both Fd and FNR in wheat (Fig. 5a).

For photosynthesis, the cytochrome b6f complex, Fd and FNR transfer electrons, whereby introducing protons into the thylakoid space to generate an electrochemical gradient that stores energy for ATP synthesis^17^. In plants, ATP synthase is integrated into thylakoid membrane. In this study, three different subunit types of ATP synthase, alpha, beta and epsilon-b, were identified as acetylprotein in wheat (Fig. 5a).

Calvin cycle, the light-independent reactions of photosynthesis, is a chemical reaction that converts carbon dioxide and other compounds into glucose. In wheat, a large proportion of metabolic enzymes involved in Calvin cycle, such as ribulose bisphosphate carboxylase/oxygenase (RuBisco), phosphoglycerate kinase (PGK), glyceraldehyde 3-phosphate dehydrogenase (GAPDH), triosephosphate isomerase (TPI), fructose-1,6-bisphosphatase (FBP), Transketolase (TK) and Sedoheputulose-1,7-bisphosphatase (SBP), were found to be acetylated (Fig. 5b).

Taken together, these findings support a notion that lysine acetylation potentially is an important part of the regulatory mechanism in photosynthesis and carbon metabolism.

### Protein interaction network of acetylated proteins in wheat

**Figure 6:**
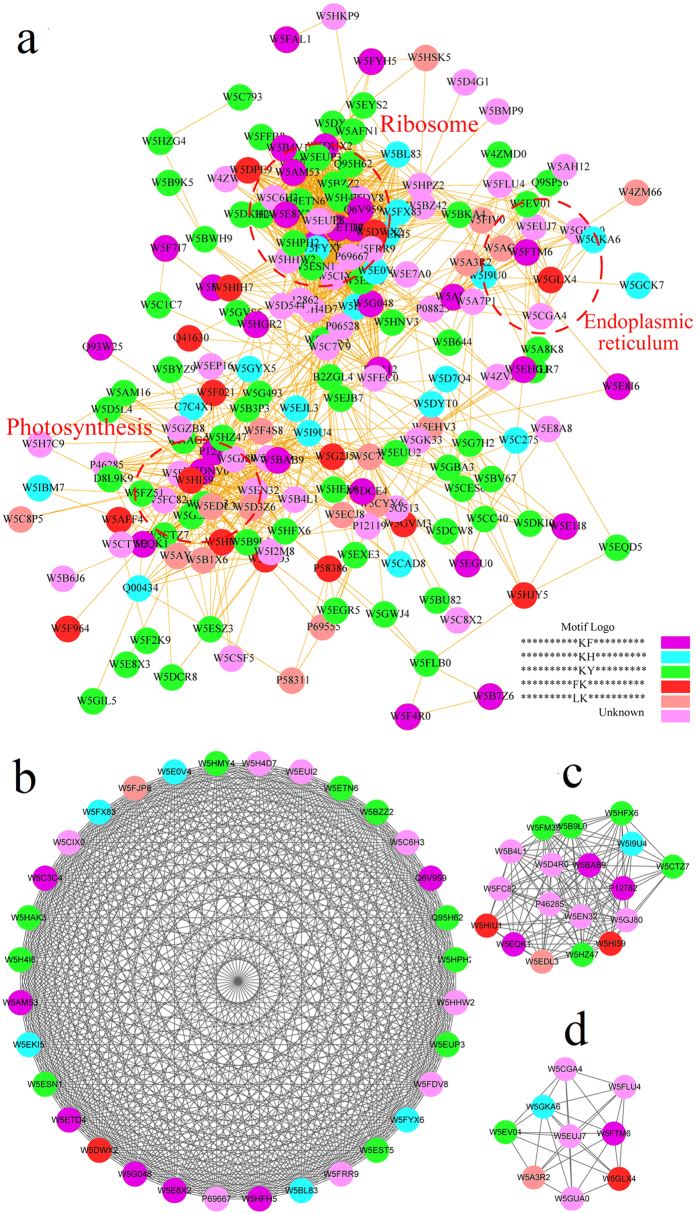
Interaction networks of the acetylated proteins in wheat. (**a**) Interaction network of all acetylated proteins. (**b**) Interaction network of acetylated proteins associated with ribosome. (**c**) Interaction network of acetylated proteins involved in photosynthesis. (**d**) Interaction network of acetylated proteins involved in endoplasmic reticulum processing.

To investigate cellular processes regulated by acetylation in wheat, the protein interaction network was established (Fig. 6). The results showed that a total of 196 acetylated proteins were mapped to the protein interaction database (Fig. 6, Supplementary Table S9, Supplementary Table S10), which presents a global view of how acetylated proteins perform diverse types of functions in wheat. According to the algorithm of Cytoscape software, eleven highly interconnected clusters of acetylated proteins were retrieved (Fig. 6a, Supplementary Table S9). The top cluster (Cluster I) identified above consists of ribosome-associated proteins, whereas Clusters II–III consist of proteins involved in photosynthesis and endoplasmic reticulum (Fig. 6a). The subnetwork graphs of these three pathways revealed that they all comprise a dense protein interaction network (Fig. 6b–d). Therefore, the physiological interactions among these protein complexes are likely to contribute to their cooperation and coordination in wheat.

## Discussion

Lysine acetylation is emerging as a widespread and highly conserved post-translational modification in both eukaryotes and prokaryotes with diverse biological functions. To date, the acetylome has been reported only in a limited number of plant species including *Arabidopsis*, grape, pea, potato, rice and soybean^18^,^19^,^20^,^21^,^22^,^23^. In this paper, we describe a proteomics study of lysine acetylation in common wheat, one of the most important crops in the world. We identified a total of 277 acetylated proteins with 416 unique modification sites through a combination of highly sensitive immuno-affinity purification and high-resolution LC−MS/MS. These acetylated proteins are localized to multiple cellular compartments and belong to diverse functional groups, suggesting that lysine acetylation plays important roles in regulating numerous cellular processes in wheat. To confirm this hypothesis, protein interaction network analysis demonstrates that a wide range of interactions are modulated by protein acetylation. These data represents the first comprehensive view of the acetylome in wheat.

In plants, one of the most important metabolic processes is photosynthesis. This process takes place in chloroplast, converting light energy to chemical energy and storing it in the bonds of sugar. Our data revealed that 100 of acetylated proteins were localized in chloroplast in wheat (Fig. 3) and many of them were involved in photosynthesis including LHC a-binding protein (Lhca1) and LHC b-binding proteins (Lhcb3, Lhcb5 and Lhcb6) (Fig. 6). In Arabidopsis, Lhcb proteins, Lhcb1 and Lhcb5, were also found to contain lysine modification sites^24^. It is well-known that LHC b-binding proteins are the major antenna protein complex for PSII in green plants, which transfer light energy to one chlorophyll a molecule at the reaction center of photosystems^25^. In cyanobacteria, the major antenna protein complexs for PSII were phycocyanin and allophycocyanin. Interestingly, a number of subunits of phycocyanin (CpcA, CpcB, CpcC, and CpcG) and allophycocyanin (ApcA, ApcB, ApcD, ApcE, and ApcF), were found to be lysine acetylated in *Synechocystis*^14^.Thus, reversible lysine acetylation may influence the function of antenna protein complex in green plants (eukaryotes) as well as in cyanobacteria (prokaryotes).

Another important function of photosynthesis is converting light energy to chemical energy which involves electron transfer and ATP synthesis. In this study, several components important for this function, including Cyt b6, Cyt f, Pet D, Fd, FNR and three subunit types of the ATP synthase, were all identified as acetylated proteins in wheat (Fig. 6). In support of our findings, the b-subunit of chloroplastic ATP synthase was found to be lysine acetylated in Arabidopsis^24^. In addition, lysine acetylation was also observed on some of the electron transfer related proteins such as FD and FNR in cyanobacterium *Synechocystis* sp.^14^. All these findings highlight the notion that lysine acetylation plays a key regulatory role in the process of photosynthesis.

As an important cereal crop in the world, wheat provides a large amount of starch ever year, which is synthesized from the raw material, starch^26^, one product of photosynthesis. In photosynthesis, Calvin cycle converts carbon dioxide and other compounds into glucose. In this research, a large proportion of metabolic enzymes in Calvin cycle were found to be acetylated in wheat (Fig. 6b). Similar results were also found in cyanobacterium^14^ and Arabidopsis^24^. Interestingly, the large chain of ribulose-bisphosphate carboxylase (RuBisco) (accession no. W5HGR2) contained 13 acetylation sites in wheat (Supplementary Table S1). Further study revealed a lysine residue was acetylated in RuBisco on Lys161 (Supplementary Table S1), which is located in the active domain in RuBisco. RuBisco is the major protein in the stroma of chloroplasts, which catalyses the carboxylation of ribulose-1,5-bisphosphate, thus fixing carbon dioxide as the first step of the Calvin cycle^27^. These studies suggest that, in both green plants and cyanobacteria, lysine acetylation is involved in regulating the activity of enzymes in Calvin cycle.

To summarize, our results provide the first extensive data on lysine acetylation in wheat. Our findings reinforce the notion that lysine acetylation plays a critical regulatory role in diverse aspects of cellular process, especially in photosynthesis and Calvin cycle. This study widens the range of physiological processes regulated by lysine acetylation and provides a rich resource that can be used to examine the functions of lysine acetylation in wheat and likely in all plants.

## Methods

### Plant material and growth conditions

Common wheat variety (*T. aestivum* L.), Chinese Spring, was used in the study and the seedlings were grown in a greenhouse with the temperature set at 22/18 °C (day/night) and a photoperiod of 16/8 h (light/dark)^28^. The leaves were excised from 3-week-old seedlings and immediately used for protein extraction.

### Protein extraction

Sample was grinded in liquid nitrogen. The cell powder was mixed with extraction buffer containing 8 M urea, 1% Triton-100, 65 mM dithiothreitol (DTT), 0.1% protease inhibitor cocktail, 3 μM trichostatin A and 50 mM nicotinamide^1^, and was sonicated three times on ice using a high intensity ultrasonic processor. The remaining debris was removed by centrifuging at 20,000 × g 4 °C for 10 min. The protein in the supernatant was precipitated with cold Trichloroacetic acid (TCA) (supernatant/TCA, 17:3, *v*/*v*) at −20 °C for 2 h. After centrifugation at 4 °C for 10 min, the precipitated protein was washed with cold acetone for three times. Finally, the protein was redissolved in buffer (8 M urea, 100 mM NH_4_HCO_3_, pH 8.0) and the protein concentration was estimated with 2-D Quant kit (GE Healthcare) according to the manufacturer’s instructions.

### Trypsin digestion

Ten mg protein was reduced with 10 mM DTT for 1 h at 37 °C and alkylated with 20 mM iodoacetamide for 45 min at room temperature in darkness. For trypsin digestion, the protein sample was diluted by adding 100 mM NH_4_HCO_3_ to urea concentration less than 2 M. Finally, the diluted protein samples were digested with trypsin at 1:50 trypsin-to-protein mass ratio for the first digestion overnight, and 1:100 trypsin-to-protein mass ratio for a second 4 h-digestion.

### Enrichment of Lys-acetylated peptides

To enrich acetylated peptides, the tryptic peptides were dissolved in NETN buffer (100 mM NaCl, 1 mM EDTA, 50 mM Tris-HCl, 0.5% NP-40, pH 8.0) followed by incubation with pre-washed agarose-conjugated anti-acetyllysine antibody beads (PTM Biolabs) at 4 °C for 12 h with gentle shaking^14^. The beads were washed four times with NETN buffer and twice with ddH_2_O and the bound peptides were eluted with 0.1% trifluoroacetic acid^14^. The eluted fractions were combined and vacuum-dried. The resulting peptides were cleaned with C18 ZipTips (Millipore) according to the manufacturer’s instructions.

### LC-MS/MS Analysis

Three parallel analyses for each fraction were performed. The enriched peptides were analyzed using mass spectrometer (Thermo Scientific^TM^ Q Exactive^TM^ Plus). Briefly, peptides were dissolved in 0.1% formic acid (FA) and directly loaded onto a reversed-phase pre-column (Acclaim PepMap 100, Thermo Scientific). Peptide separation was performed using a reversed-phase analytical column (Acclaim PepMap RSLC, Thermo Scientific) at a constant flow rate of 300 nl/min on an EASY-nLC 1000 UPLC system. The gradient was comprised of an increase from 7% to 9% solvent buffer (0.1% FA in 98% acetonitrile) for 2 min, 9% to 24% for 24 min, 24% to 35% for 8 min and climbing to 80% in 3 min then holding at 80% for the last 3 min. The resulting peptides were subjected to NSI source followed by tandem mass spectrometry (MS/MS) in Q ExactiveTM Plus (Thermo) coupled online to the UPLC. Intact peptides were detected in the Orbitrap at a resolution of 70,000. Peptides were selected for MS/MS using NCE setting as 30; ion fragments were detected in the Orbitrap at a resolution of 17,500. A data-dependent procedure that alternated between one MS scan followed by 20 MS/MS scans was applied for the top 20 precursor ions above a threshold ion count of 1.5E4 in the MS survey scan with 30.0 s dynamic exclusion. The electrospray voltage applied was 2.0 kV. Automatic gain control was used to prevent overfilling of the ion trap; 5E4 ions were accumulated for generation of MS/MS spectra. For MS scans, the m/z scan range was 350 to 1800^29^.

### Data Analysis

The MS/MS data obtained above were processed using MaxQuant with integrated Andromeda search engine (v.1.4.1.2)^30^,^31^. Tandem mass spectra were searched against UniProt_*Wheat* (100,981 sequences) database concatenated with reverse decoy database. Peptide sequences were searched using trypsin specificity^32^ and allowing to 4 missing cleavages, 5 modifications per peptide and 5 charges. Mass error was set to 20 ppm for first search, 5 ppm for main search and 0.02 Da for fragment ions. Carbamidomethylation on Cys was specified as fixed modification and oxidation on Met, acetylation on Lys and acetylation on protein N-terminal were specified as variable modifications. False discovery rate thresholds for protein, peptide and modification site were specified at 1%^14^,^33^. Minimum peptide length was set at 7. All the other parameters in MaxQuant were set to default values. The site localization probability was set as >0.75.

### Bioinformatics analysis

Soft motif-x was used to analyze the model of sequences constituted with amino acids in specific positions of modifier-21-mers (10 amino acids upstream and downstream of the acetylation site) in all acetylated protein identified. Secondary structures of proteins were predicted by NetSurfP^34^. The probability for one given type of secondary structure of the modified lysine residues was compared to those of the control residues for all acetylated proteins identified in this study, and *p* value was calculated as previously described^35^. Gene Ontology (GO) annotation proteome was derived from http://www.ebi.ac.uk/GOA and the proteins were classified by GO annotation based on three categories: biological process, cellular component and molecular function. Kyoto Encyclopedia of Genes and Genomes (KEGG) database was used to annotate protein pathway^36^. Functional annotation tool of DAVID bioinformatics resources 6.7 was used to identify GO term and KEGG pathway^37^. A two-tailed Fisher’s exact test was employed to test the enrichment of the protein-containing IPI entries against all IPI proteins. Correction for multiple hypothesis testing was carried out using standard false discovery rate control methods. The GO and pathway with a corrected *p*-value < 0.05 were considered significant. WoLF PSORT, a subcellular localization predication program, was used to predict subcellular localization of the identified proteins. Protein-protein interactions for the identified acetylated proteins were performed using Cytoscape software and the protein-protein interaction network was obtained from the STRING database according to the methods described previously^38^,^39^.

## Additional Information

**How to cite this article**: Zhang, Y. *et al.* Comprehensive profiling of lysine acetylproteome analysis reveals diverse functions of lysine acetylation in common wheat. *Sci. Rep.* **6**, 21069; doi: 10.1038/srep21069 (2016).

## Supplementary Material

Supplementary Figures

Supplementary Dataset S1

Supplementary Dataset S2

Supplementary Dataset S3

Supplementary Dataset S4

Supplementary Dataset S5

Supplementary Dataset S6

Supplementary Dataset S7

Supplementary Dataset S8

Supplementary Dataset S9

Supplementary Dataset S10

## Figures and Tables

**Table 1:** Comparison of *T. aestivum* L. acetylome with other published acetylome in plants.

Plant	No. of acetylation sites	No. of acetylated proteins	Reference
*T. aestivum* L.	416	277	This study
*Oryza sativa*	60	44	20
*Arabidopsis thaliana*	91	74	39
*Glycine max*	190	121	23
*Pisum sativum*	664	358	22
*Vitis vinifera*	138	97	18
*Solanum tuberosum*	35	31	21
