# Supplementary material for: Comprehensive profiling of lysine acetylproteome analysis reveals diverse functions of lysine acetylation in common wheat
Source: Sci Rep. 2016 Feb 15;6:21069. doi: 10.1038/srep21069 (PMC4753473; doi:10.1038/srep21069)

**Comprehensive profiling of lysine acetylproteome analysis reveals diverse functions of lysine acetylation in common wheat**

Yumei Zhang^1,2,†^, Limin Song^2,†^, Wenxing Liang^2^, Ping Mu^2^, Shu Wang^1,*^ & Qi Lin^2,*^

^1^College of Agronomy, Shenyang Agricultural University, Shenyang, Liaoning 110866, China. ^2^College of Agronomy and Plant Protection, Qingdao Agricultural University, Qingdao, Shandong 266109, China. ^†^These authors contributed equally to this work. ^*^Correspondence and requests for materials should be addressed to S.W. (wangshusl@126.com) or Q.L. (nxxlinqi@163.com)

**Figure S1.** KEGG pathway enrichment analysis of the acetylated proteins in carbon metabolism. The acetylated proteins are in yellow.


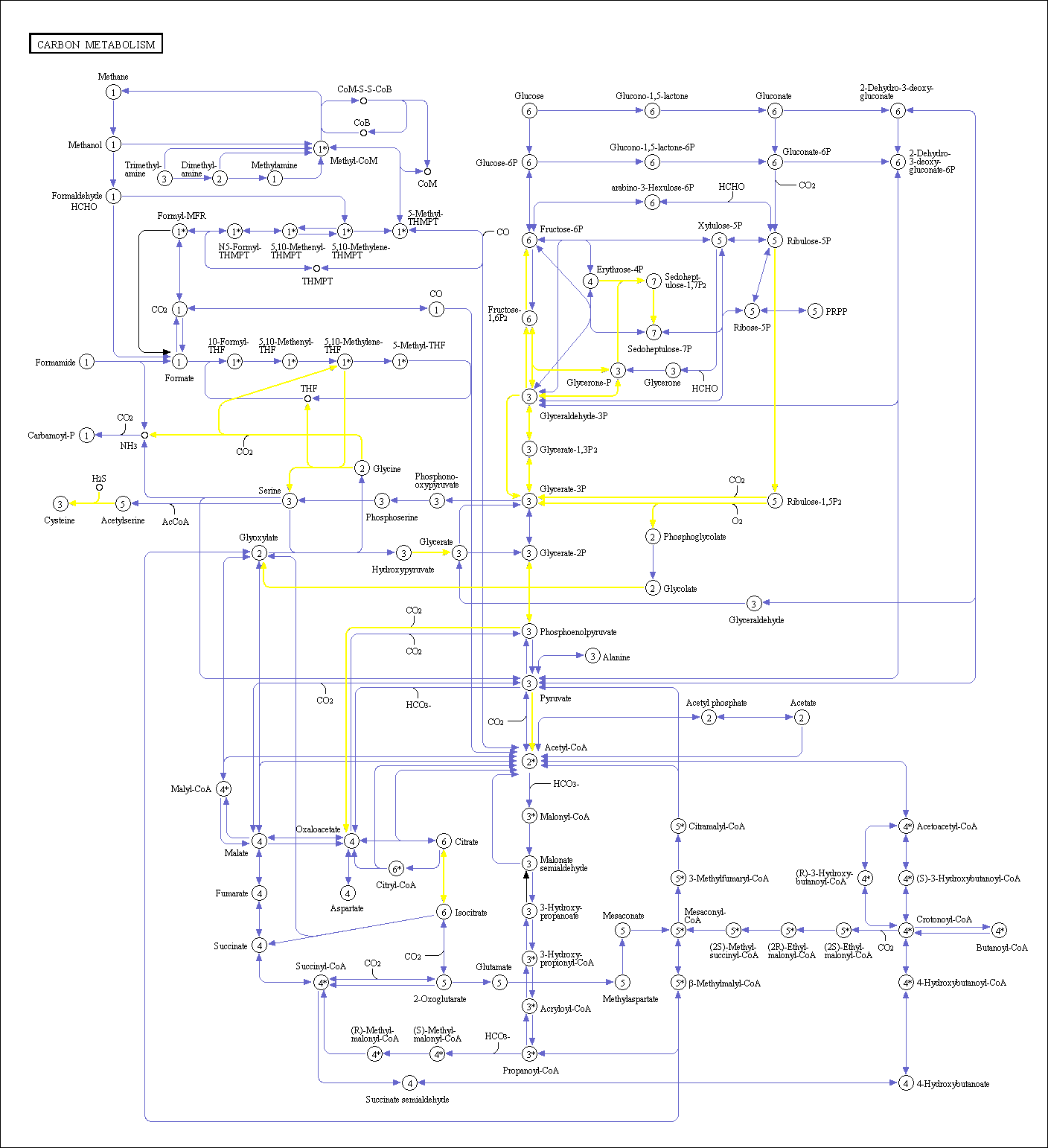


**Figure S2.** KEGG pathway enrichment analysis of the acetylated proteins in glycolysis and gluconeogenesis. The acetylated proteins are in yellow.

**
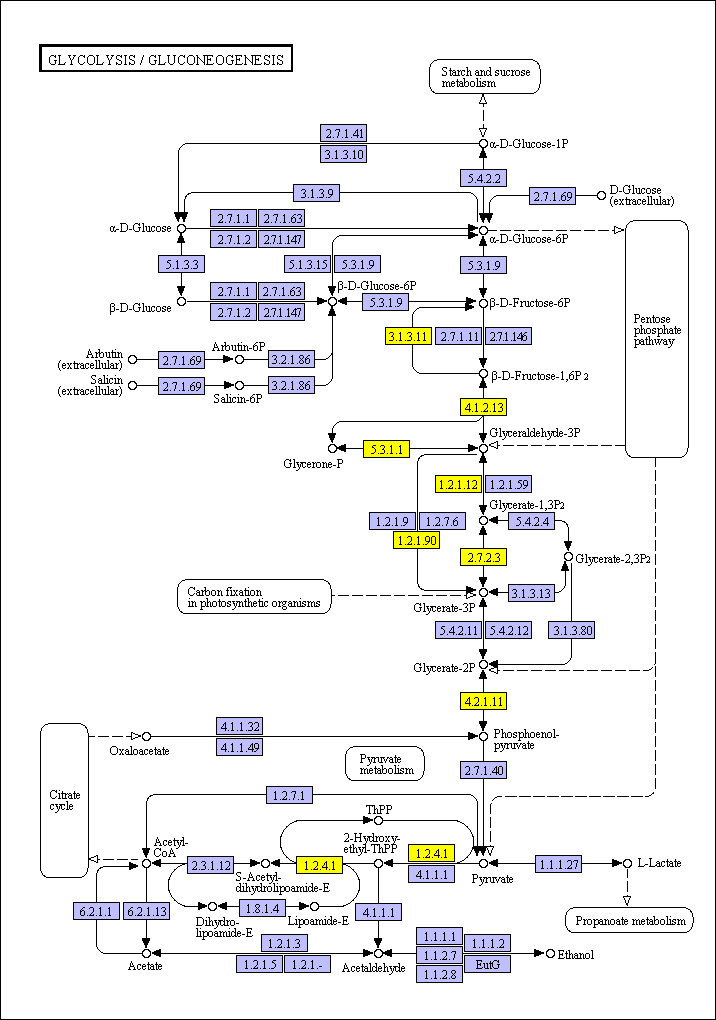
**

**Figure S3.** KEGG pathway enrichment analysis of the acetylated proteins in carbon fixation in photosynthetic organisms. The acetylated proteins are in yellow.


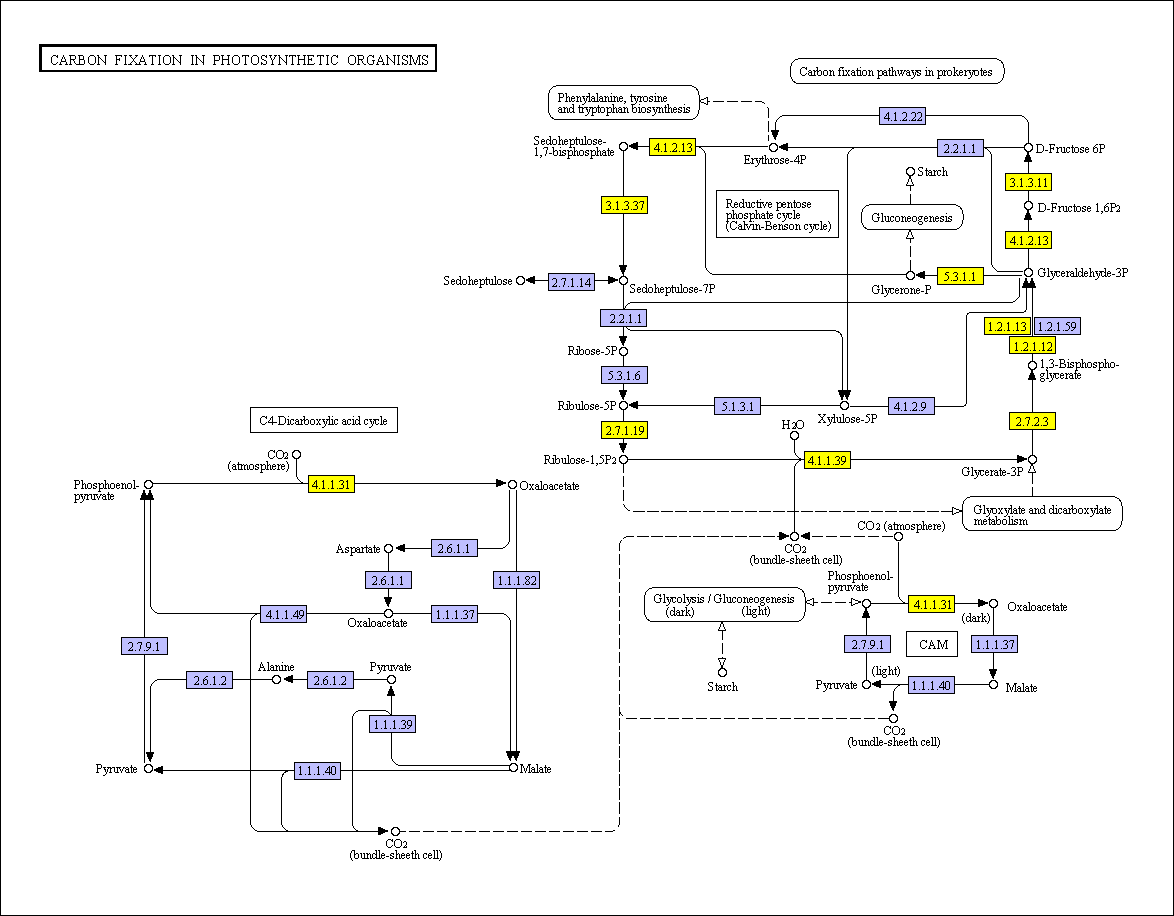


**Figure S4.** KEGG pathway enrichment analysis of the acetylated proteins in biosynthesis of amino acids. The acetylated proteins are in yellow.


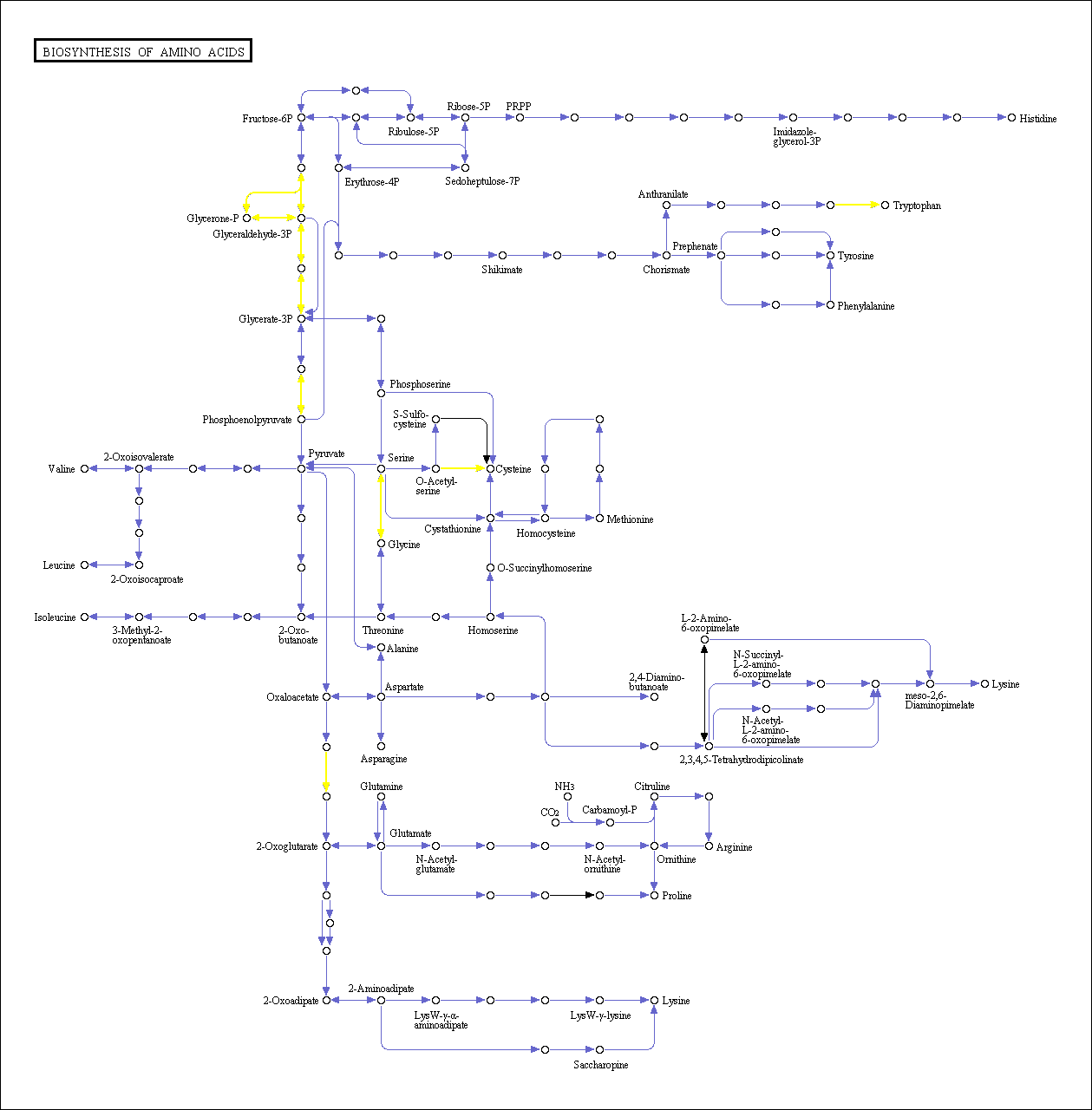

Supplement: Supplementary Figures [file srep21069-s1.doc]
